# Supplementary figures and images for: Raman spectral analysis of microbial pigment compositions in vegetative cells and heterocysts of multicellular cyanobacterium
Source: Biochem Biophys Rep. 2023 Apr 14;34:101469. doi: 10.1016/j.bbrep.2023.101469 (PMC10133670; doi:10.1016/j.bbrep.2023.101469)

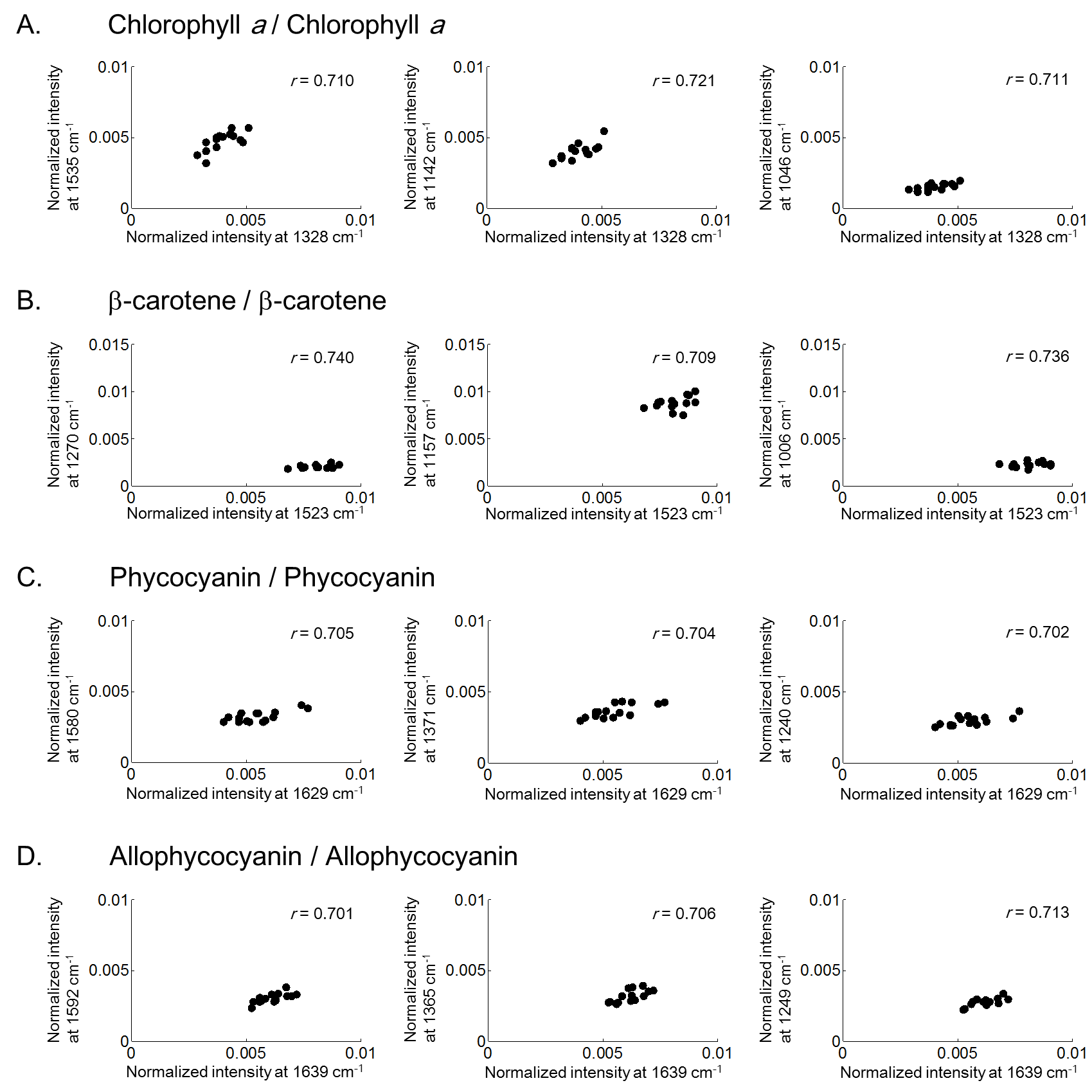


Figure S1

Supplement: Multimedia component 1 [file mmc1.docx]
